# Microtubule destabilization caused by silicate via HDAC6 activation contributes to autophagic dysfunction in bone mesenchymal stem cells

**DOI:** 10.1186/s13287-019-1441-4

**Published:** 2019-11-27

**Authors:** Zheng Li, Shuhao Liu, Tengfei Fu, Yi Peng, Jian Zhang

**Affiliations:** Department of Orthopaedics, Zhongshan Hospital of Fudan University, 180 Fenglin Road, Shanghai, China

**Keywords:** BMSCs, Silicate, Autophagic flux, Microtubule, HDAC6

## Abstract

**Background:**

Silicon-modified biomaterials have been extensively studied in bone tissue engineering. In recent years, the toxicity of silicon-doped biomaterials has gradually attracted attention but requires further elucidation. This study was designed to explore whether high-dose silicate can induce a cytotoxicity effect in bone mesenchymal stem cells (BMSCs) and the role of autophagy in its cytotoxicity and mechanism.

**Methods:**

Morphologic changes and cell viability of BMSCs were detected after different doses of silicate exposure. Autophagic proteins (LC3, p62), LC3 turnover assay, and RFP-GFP-LC3 assay were applied to detect the changes of autophagic flux following silicate treatment. Furthermore, to identify the potential mechanism of autophagic dysfunction, we tested the acetyl-α-tubulin protein level and histone deacetylase 6 (HDAC6) activity after high-dose silicate exposure as well as the changes in microtubule and autophagic activity after HDAC6 siRNA was applied.

**Results:**

It was found that a high dose of silicate could induce a decrease in cell viability; LC3-II and p62 simultaneously increased after high-dose silicate exposure. A high concentration of silicate could induce autophagic dysfunction and cause autophagosomes to accumulate via microtubule destabilization. Results showed that acetyl-α-tubulin decreased significantly with high-dose silicate treatment, and inhibition of HDAC6 activity can restore microtubule structure and autophagic flux.

**Conclusions:**

Microtubule destabilization caused by a high concentration of silicate via HDAC6 activation contributed to autophagic dysfunction in BMSCs, and inhibition of HDAC6 exerted a cytoprotection effect through restoration of the microtubule structure and autophagic flux.

## Background

Bone mesenchymal stem cells (BMSCs), which are derived from the bone marrow, have the capacity for multidirectional differentiation within special culture conditions [1, 2]. BMSCs play an important role in the process of bone growth, development, and repair and are indispensable to bone formation. BMSCs act both as an important source of osteoblasts and in the synthesis and secretion of various growth factors [3]. Silicate-doped biomaterials can induce the differentiation of BMSCs and enhance bone formation in a certain range [4–6]. In recent years, the cytotoxicity of silicate-doped biomaterials has gradually attracted attention, and studies have found that silicate-doped bioceramics could promote the caspase-dependent apoptosis of macrophages via altering the ionic microenvironment between the implants and hosts [7]. In clinical practice, it was found that primary total hip arthroplasty (THA) using bioactive bone cement (SiO_2_ 34.0%) showed an early radiological loosening after long-term follow-up, and the mechanism still remained unclear [8]; several researches identified that intracortical silicon microelectrode implants could cause blood-cerebral barrier dysfunction and neuronal cell loss [9, 10]. Moreover, studies have confirmed that a high concentration of silicate could inhibit the viability of human BMSCs [11]. Our previous study also identified that a high concentration of silicate could induce autophagic flux blockage and cellular apoptosis in human umbilical vein endothelial cells [12]. However, whether silicate has a cytotoxic effect on BMSCs and its mechanism remains to be further studied. Furthermore, silicon ion concentrations in different biomaterials range from 0.03 mM at the lowest up to 50 mM at the highest [13]. Silicon is a trace element in the human body, and the silicon content of most implants is significantly higher than the normal range of the human body; the potential toxicity of silicate cannot be ignored, and its logical range in BMSCs still needs further identification [13, 14].

Autophagy is a functionally and evolutionarily preserved process that degrades and recycles harmful proteins or injured organelles in eukaryotic cells [15]. Autophagy broadly includes macroautophagy, microautophagy, and chaperone-mediated autophagy. This study mainly focuses on macroautophagy, which is also the most studied. Autophagy is an adaptive response to maintain cell homeostasis and survival in the face of adverse environmental threats or stress. Disruption of autophagy can induce cells to self-repair disorders and further fall into apoptosis or necrosis [16, 17]. Moreover, Yang et al. have found that activation of autophagy could partially reverse the aging of BMSCs and increase osteogenic differentiation capacity; furthermore, autophagy could maintain the osteogenic differentiation ability of mesenchymal stem cells under adverse conditions [18, 19].

At present, there is no systematic report on the effect of soluble silicate on autophagy. Although some studies have pointed out that silicon nanoparticles can block autophagy flux by affecting the lysosome function, it is almost always caused by the structure of the nanomaterials themselves [20–22]. Whether autophagy is related to silicon ions remains to be further studied. Here, based on the treatment of BMSCs with different concentrations of sodium metasilicate representing silicon ions, the effect of free silicate on autophagy was determined through the detection of autophagy flux. Simultaneously, a reference was provided to clarify the reasonable and safe range of silicon ions in BMSC-related biomaterials.

## Methods

### Reagents and antibodies

Chloroquine (CQ) was purchased from Sigma-Aldrich (St. Louis, MO, USA). Bafilomycin A1 (Baf A1) was purchased from Santa Cruz Biotechnology (Santa Cruz, CA, USA). Cell Counting Kit-8 (CCK-8) was purchased from Dojindo Molecular Technologies (Shanghai, China). LC3 (#3868), HDAC6 (#7612), acetyl-α-tubulin (#5335), and α-tubulin (#2125) primary antibodies were purchased from Cell Signaling Technology (Danvers, MA, USA). SQSTM1/p62 (#ab56416), LAMP1 (#ab24170), and LAMP2 (#ab13524) primary antibodies were purchased from Abcam (Cambridge, UK). GAPDH (#60004-1-lg), VAMP8 (#15546-1-AP), SNAP29 (#12704-1-AP), and STX17 (#17815-1-AP) primary antibodies were purchased from Proteintech Group (Boston, MA, USA). The second antibodies (peroxidase-conjugated goat anti-rabbit IgG and peroxidase-conjugated goat anti-mouse IgG) were purchased from Yeasen Biotechnology (Shanghai, China). The sodium metasilicate solution was prepared as previously reported at a stock solution of high concentration (100 mM) with some modification [23, 24]. In brief, sodium metasilicate solution (Sigma-Aldrich, #338443) was diluted into alpha-MEM to prepare a stock solution. The pH was adjusted using HCl to a physiological range (pH about 7.4) before 10% fetal bovine serum (FBS; ScienCell, Carlsbad, CA, USA) was added.

### Cell culture and treatment

BMSCs were harvested and identified according to our previous studies [25]. In brief, the cells were extracted from the bone marrow of rats less than a month old and were identified with cell surface marker such as CD73, CD90, and CD105 as well as multilineage differentiation ability (osteogenesis, adipogenesis, and chondrogenesis). The cells were cultured and purified in alpha-MEM (Keygentec, Nanjing, China), which contained 10% FBS and 1% penicillin/streptomycin solution (P/S), in an incubator at 37 °C with 5% CO_2_. Cell dissociation and passages were performed for 3 days each time. Primary BMSCs are prone to aging, and we generally only use the cells in four generations. The medium was replaced with different concentrations of chemicals when the cells achieved 80% density and continued to culture for 24 h. Cells were pretreated with CQ or Baf A1 for 2 h and continued in the following 24 h in the corresponding group.

### ICP-AES

Inductively coupled plasma atomic emission spectrometry (ICP-AES, NexION 300X ICP-MS, PerkinElmer, Waltham, MA, USA) was applied to evaluate the cellular silicon level. BMSCs were treated with various concentrations (0, 0.1, 0.5, 1.5 mM) of silicate for 24 h. Cell samples were collected and washed with phosphate-buffered saline (PBS) twice and subsequently resuspended in PBS for cell counting. After that, the cells were centrifuged and lysed with 5 ml RIPA overnight at room temperature. The silicon standard series was prepared, and the silicon level in the samples was measured by ICP-AES. The results are presented as the silicon level in each 10^6^ cells (umol/10^6^ cells).

### Cell viability

Morphologic changes in BMSCs under the microscope (× 40) were recorded and compared after being treated with different concentrations of silicate (0, 0.1, 0.5, 1.5, 3, 5 mM). CCK-8 was used to evaluate cell viability at different time points (6 h, 24 h, 72 h). In brief, BMSCs were cultured in a 96-well plate with a density of 1 × 10^5^, and they were treated with different concentrations of silicate (0.1, 0.5, 1.5, 3, 5 mM). When the cells achieved 80% density, each group was set with 6 repeat wells. To each well, 10 μl of CCK-8 was added and cells were incubated at 37 °C for 2 h. The absorbance values were measured at a 460-nm wavelength with a spectrophotometer. The viability rate was calculated using the following formula:

Viability rate (%) = (OD treatment group − OD blank group) / (OD control group − OD blank group) × 100%

### Western blot analysis

Western blot was conducted as reported previously [12, 25]. In brief, RIPA with protease inhibitor (PMSF) was applied to lyse BMSCs on ice. A total of 30 μg protein was loaded and separated with 12.5% sodium dodecyl sulfate–polyacrylamide gel electrophoresis (12.5%; Epizyme Biotech, Shanghai, China) and then transferred to a polyvinylidene fluoride membrane (0.2 μm; Millipore, Darmstadt, Germany). The membrane was blocked with 5% nonfat milk for 1 h at room temperature and incubated with primary antibodies at 4 °C for the night. Primary antibodies, including LC3, p62, HDAC6, acetyl-α-tubulin, α-tubulin, VAMP8, SNAP29, and STX17, were diluted at 1:1000; LAMP1 and LAMP2 primary antibodies were diluted at 1:500; and GAPDH antibody was diluted at 1:5000. The secondary antibodies (HRP-1inked rabbit or mouse at 1:3000 dilution) were applied at room temperature for 2 h after TBST washing. Enhanced chemiluminescence system reagent was applied for imaging exposure, and results of the images were analyzed by Quantity One software. The experiments were repeated at least four times for each protein.

### Immunofluorescence assay

Immunofluorescence assay was performed as previously reported with some modifications [12]. For LC3 staining, the slide of the BMSCs was fixed in 4% paraformaldehyde for 10 min and subsequently permeabilized in 0.25% Triton X-100 for 15 min. After that, the slide was blocked with 3% bovine serum albumin for 30 min at room temperature and then incubated with rabbit anti-LC3 primary antibody (1:200, Cell Signaling Technology) overnight at 4 °C. Then, the cell slice was incubated in FITC-AffiniPure Goat Anti-Rabbit IgG (1:50, Yeasen Biotechnology, Shanghai, China) for 1 h at room temperature and subsequently with DAPI for 5 min. The slide was reviewed and recorded under a fluorescence microscope (Nikon, Tokyo, Japan). LC3 puncta indicated autophagosomes; the number of LC3 puncta was counted and compared in 30 random fields (× 400), and the data were presented as LC3 dots count/cell. Moreover, specific BMSCs with stable expression of the mRFP-GFP-LC3 were constructed; BMSCs were inoculated with Ad-mRFP-GFP-LC3 (#HB-AP210, Hanbio, Shanghai, China) for 48 h and then treated with corresponding reagents. The samples were reviewed and recorded under a fluorescence microscope. For the lyso-tracker, BMSCs were cultured in a 12-well plate and divided into the following groups: control group, silicate (3 mM)-treated group, Baf A1 (100 nM) or CQ (20 uM) group. Lyso-tracker solution (Keygentec, Nanjing, China) was diluted in alpha-MEM at 1:500 and co-cultured with BMSCs at 37 °C for 30 min. The slide was reviewed and recorded under a fluorescence microscope (Nikon, Tokyo, Japan). The fluorescence intensity was evaluated and analyzed using ImageJ software in 30 random cells, and the average fluorescence intensity per cell was compared. For the tubulin-tracker assay, BMSCs were cultured in slides and treated with different concentrations of silicate or interference for 24 h in the corresponding group. The slide of BMSCs was fixed in 4% paraformaldehyde for 10 min and subsequently washed twice in 0.1% Triton X-100 for 5 min. The tubulin-tracker solution (Keygentec, Nanjing, China) was diluted in alpha-MEM in 1:200 and co-cultured with BMSCs at 37 °C for 1 h. The slide was reviewed and compared via laser scanning confocal microscopy (Leica, Wetzlar, Germany).

### Real-time quantitative polymerase chain reaction

Total RNA from treated cells was extracted via RNA Extraction Reagent (Yeasen Biotech, Shanghai, China) according to the manufacturer’s instructions. The concentration and quality of RNA were evaluated by spectrophotometric determination at 260/280 nm, and equal amounts of RNA from each sample were used for cDNA reverse-transcription following the PrimeScript RT Reagent Kit with gDNA Eraser (Takara Biomedical Technology, Beijing, China). cDNA was further applied as a template for real-time quantitative polymerase chain reaction (RT-qPCR) following the manufacturer’s instructions (Mastercycler Gradient, Eppendorf, Germany). The primers were listed as follows:

Syntaxin 17 (STX17): 5′-GAGGGTCCGTCAGTCAAGTT-3′ (forward), 5′-CTGACCCTCAGGCATCCAAT-3′ (reverse)

Synaptosome associated protein 29 (Snap29): 5′-CCCTTCCTGCTTCCAAGGTT-3′ (forward), 5′-CCCTGCGTAACACCTCTTGT-3′ (reverse)

Vesicle-associated membrane protein 8 (Vamp8): 5′-GGAAGCCACGTCTGAACACTT-3′ (forward), 5′-GATGGTGCCCGTAGCAAAGA-3′ (reverse).

### HDAC6 activity assay

HDAC6 activity assay was conducted according to the manufacturer’s instructions (# 50076, BPS Bioscience, San Diego, CA USA). In brief, cell lysates of BMSCs were diluted in an HDAC assay buffer and subsequently mixed with the substrate. After that, a HDAC developer was added and the cell lysates continued to incubate. The values were measured with a spectrofluorometer with excitation at a wavelength of 380 nm and detection of emitted light of 460 nm.

### SiRNA transfection

The cells of each group were inoculated in 6-well plates 1 day before transfection. Alpha-MEM free of antibiotics was used for transfection dilution. The primer sequence of siRNA-HDAC6 (siHDAC6, RiboBio Co. Guangzhou, China) was designed as follows:

Sense: 5′-GCCGUAUUAUUCUUAUUCUdTdT-3′

Antisense: 5′-AGAAUAAGAAUAAUACGGCdTdT-3′

SiHDAC6 and its negative control sequences were mixed with Lipofectamine 2000 (Invitrogen, Carlsbad, CA, USA) according to the manufacturer’s instructions. Following a 20-min incubation at 37 °C, the mixture was added to plates and continued to culture for 4–6 h and then used in subsequent experiments (nsRNA: vehicle group and silicate group (3 mM); SiHDAC6: vehicle group and silicate group (3 mM)). The transfection efficiency was calculated according to the mRNA expression of HDAC6 after the cells were treated with 50 nM siRNA for 48 h.

### Statistical analysis

Data are presented as mean ± standard deviation (SD) and analyzed with GraphPad Prism 5.0 software (San Diego, CA, USA) or SPSS 20.0 (IBM Corp., Armonk, NY, USA). Multigroup comparisons of the means were carried out by one-way analysis of variance (ANOVA) test with post-test and by Student–Newman–Keuls (S-N-K) multiple comparison test, and *p* < 0.05 indicated statistical significance.

## Results

### High concentration of silicate affected morphologic changes and cell viability of BMSCs

**Fig. 1:**
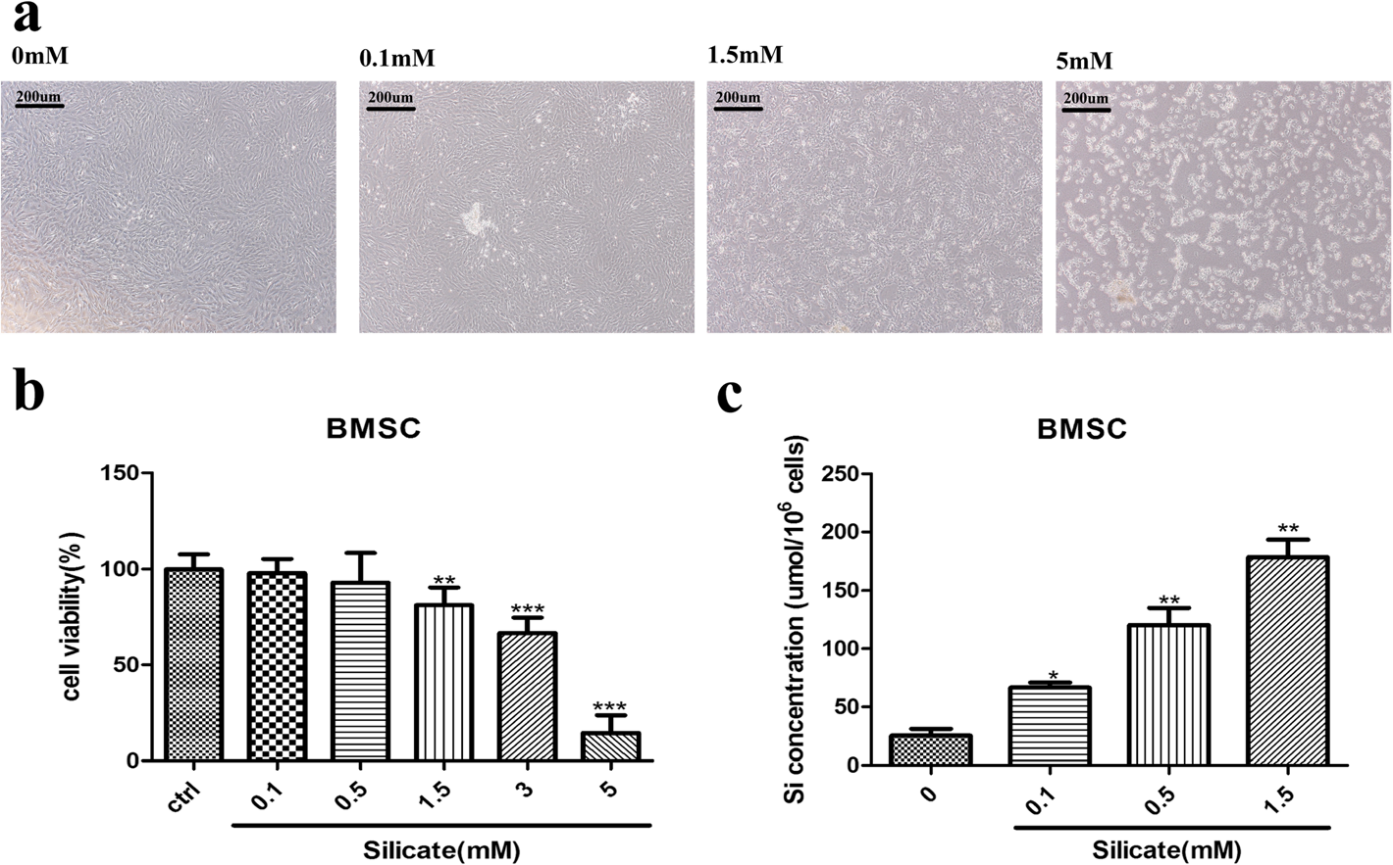
High concentration of silicate affected morphologic changes and cell viability of BMSCs. **a** Morphologic changes (× 40) of BMSCs after treatment with different concentrations of sodium metasilicate (0, 0.1, 1.5, and 5 mM). As silicate concentrations increased, the number of suspended and deactivated cells increased. **b** CCK-8 was applied to detect cell viability (*n* = 6) after treatment with different concentrations (0, 0.1, 0.5, 1.5, 3, 5 mM) of silicate. Cell viability decreased significantly in groups treated with greater than 1.5 mM silicate compared with that in the control group. **c** Intracellular silicon concentration increased with extracellular concentration (*n* = 4). (*p < 0.05, **p < 0.01, ***p < 0.001)

Figure 1a displays the morphologic changes (× 40) of BMSCs after treatment with different concentrations of sodium metasilicate (0, 0.1, 0.5, 1.5, 3, 5 mM). As the concentration of sodium metasilicate increased, the number of suspended and deactivated cells increased. CCK-8 was applied to detect cell viability (*n* = 6) after treatment with different concentrations (0, 0.1, 0.5, 1.5, 3, 5 mM) of silicate at different time points (6 h, 24 h, 72 h). As we found, the cell viability decreased significantly after 3 mM silicate (0.80 ± 0.07) exposure for 6 h compared with that of the control group (1.01 ± 0.07) (Additional file 1 a). After the 24-h silicate treatment, the cell viability in the group treated with 1.5 mM (0.81 ± 0.04) was lower than that of the control group (0.99 ± 0.04, *p* < 0.01). The value continued to decrease as the concentration of sodium metasilicate increased (*p <* 0.001); especially in the 5 mM group (0.14 ± 0.04), most cells lost their viability under a high concentration of silicon, whereas in the 0.1 mM group (0.98 ± 0.03) and 0.5 mM group (0.93 ± 0.07), cell viability showed no significant difference compared with that of the control group. After the 72-h silicate exposure, the cell viability in the group treated with 1.5 mM silicate further decreased (0.40 ± 0.05), while the cell viability still showed no significant change in the 0.1 mM group (1.03 ± 0.08) and 0.5 mM group (0.97 ± 0.06) (Additional file 1 b). To further confirm whether low concentration of silicate (0.1 mM and 0.5 mM) have cytotoxicity on BMSCs, the morphologic changes of BMSCs were recorded after 3 weeks of treatment with 0.1 mM and 0.5 mM silicate. Compared with the control group, the silicate did not show obvious cytotoxicity, the cells did not float or break as they did after treatment with high concentration of silicate, and some nodular changes can be observed during cell proliferation (arrow) (Additional file 2 a–c). ICP-AES was used to detect the concentration of silicon inside the cells after treatment with different concentrations of silicate (0, 0.1, 0.5, 1.5 mM) for 24 h. As shown in Fig. 1c, the concentration of silicon inside the cells increased significantly with extracellular concentration.

### High concentration of silicate impaired autophagic flux in BMSCs

**Fig. 2:**
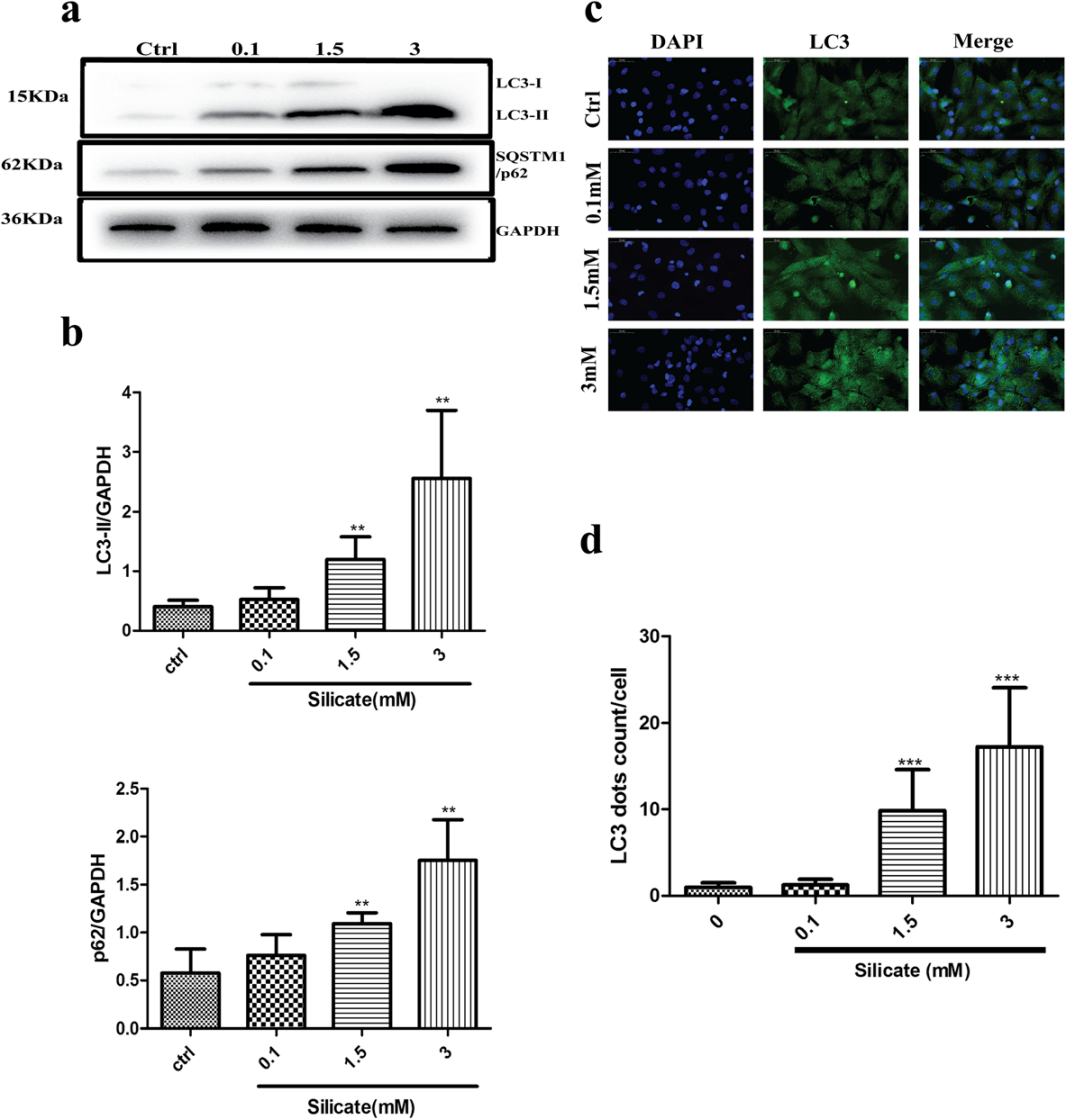
Autophagosomes accumulated in high concentration of silicate-treated BMSCs. **a, b** Western blot showed the expression of LC3-II and p62 protein after silicate exposure for 24 h (*n* = 4). LC3-II and p62 simultaneously increased significantly in groups treated with more than 1.5 mM silicate as compared with that in the control group. **c, d** Autophagosomes were largely accumulated in BMSCs treated with greater than 1.5 mM silicate from LC3 fluorescence (*n* = 3, 10 random fields per sample). (**p < 0.01, ***p < 0.001)

**Fig. 3:**
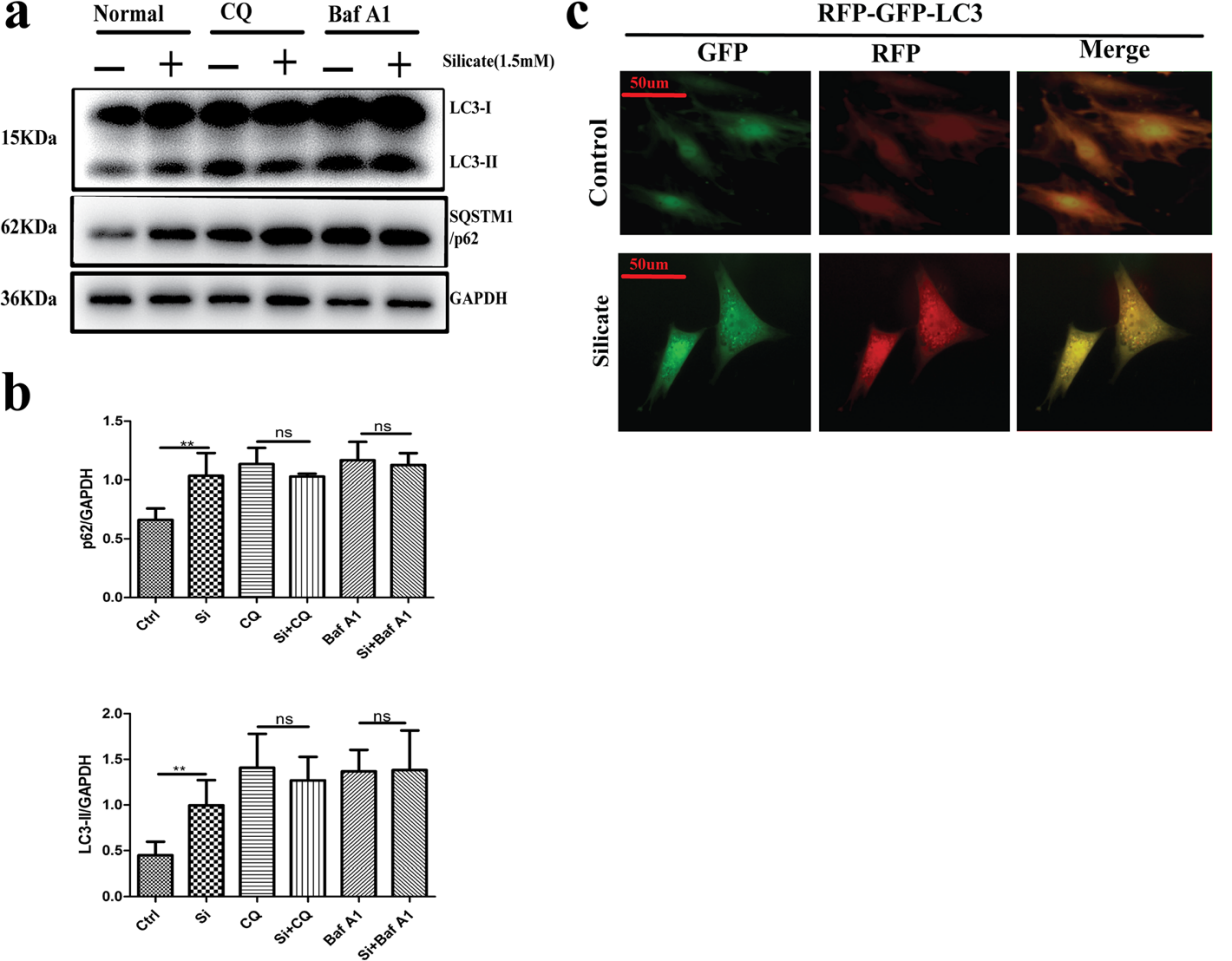
High dose of silicate exposure blocked the fusion of autophagosomes with lysosomes. **a, b** Western blot was performed to characterize the expression of LC3 and SQSTM1/p62 in BMSCs pretreated with Baf A1 (100 nM) and CQ (20 uM) for 2 h before exposure to 1.5 mM of silicate for 24 h (*n* = 4). The data indicated no significant difference after Baf A1 and CQ co-treatment. **c** mRFP-GFP-LC3 expression demonstrated that autophagosomes were largely accumulated in BMSCs after exposure to 1.5 mM silicate (yellow puncta), and the fusion of autophagosomes with lysosomes was blocked. (**p < 0.01, ns: no significance)

The simultaneous increase in LC3-II and SQSTM1/p62 always indicated a blockage of autophagic flux. BMSCs were treated with different concentrations of silicate (0, 0.1, 1.5, 3 mM) for 24 h, and Western blot was used to detect the protein expression of LC3-II and p62. As shown in Fig. 2a, b, data analysis demonstrated that LC3-II and p62 expression increased significantly (*p* < 0.01) in the 1.5 mM and 3 mM groups compared with that of the control group. The immunofluorescence of LC3 (Fig. 2c) further confirmed that autophagosomes (LC3-positive dots) largely accumulated in BMSCs after treatment with 1.5 mM or 3 mM silicate, and data analysis (Fig. 2d) showed that the number of LC3 dots in the 1.5 mM and 3 mM groups significantly increased (*p* < 0.001) compared with that of the control group. To further confirm the blockage of autophagic flux, an LC3 turnover assay was applied. Baf A1 and CQ, which are classic inhibitors of the autophagy pathway, were introduced to observe LC3 conversion. As shown in Fig. 3a, b, the protein expression of LC3-II and p62 was significantly increased (*p* < 0.01) after treatment with 1.5 mM silicate compared with that of the control group; however, there was no significant difference in LC3-II and p62 expression after co-treatment with Baf A1 or CQ. Figure 3c displays the mRFP-GFP-LC3 expression after silicate exposure. Red dots indicate autolysosomes, while yellow dots indicate autophagosomes. As shown, autophagosomes were largely accumulated in BMSCs after exposure to 1.5 mM silicate (yellow puncta), and the fusion of autophagosomes with lysosomes was blocked. Moreover, to further confirm the effects of silicate on autophagy, a low concentration of silicate (0.1 mM and 0.5 mM) was used to treat the BMSCs and we analyzed the expression of LC3-II and p62 proteins via Western blot after a long-term culture of BMSCs. It was also found that there was no statistical difference in protein changes (*n* = 4), which indicated that BMSCs had good tolerance to a low concentration of silicate and the mechanism reaction was different from that of a high concentration of silicate (Additional file 2 d–e).

### Silicate impaired autophagic flux via disrupting microtubule stability

**Fig. 4:**
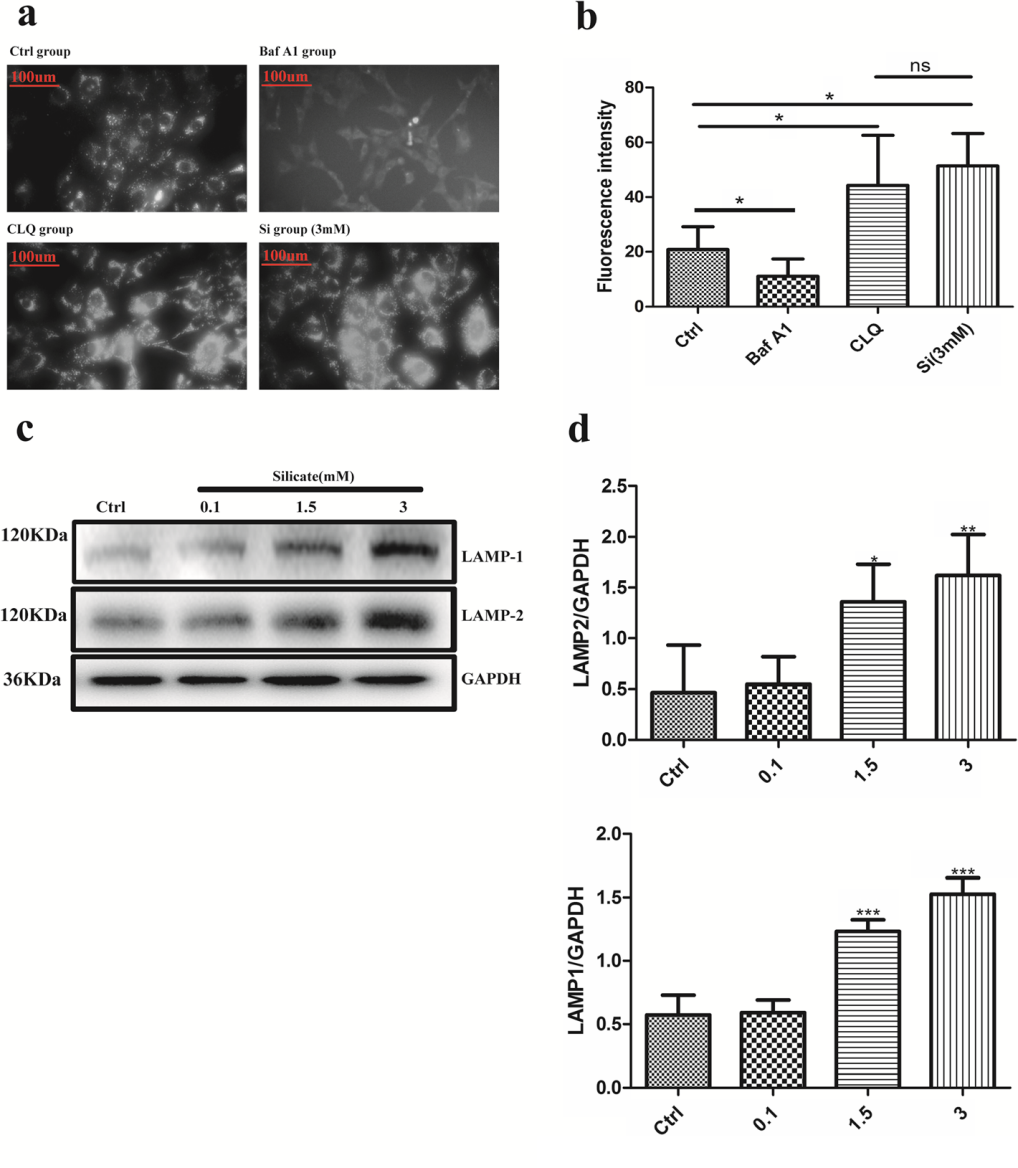
High concentration of silicate did not affect lysosome function of BMSCs. **a, b** Lyso-tracker assay, which could lock acid lysosomes, was applied to evaluate the function of lysosomes, and the Baf A1 and CQ treatment groups were set as the positive control groups (*n* = 3, 30 random cells per sample). The data indicated that the fluorescence intensity in the silicate (3 mM)-treated groups was similar to that of the CQ-treated group, and the intensity increased significantly compared with that of the control group. **c, d** Western blot was performed to characterize the expression of LAMP1 and LAMP2 in BMSCs after exposure to silicate for 24 h (*n* = 4). Data analysis showed that LAMP1 and LAMP2 increased significantly in the high-concentration silicate group. (*p < 0.05, **p < 0.01, ***p < 0.001)

**Fig. 5:**
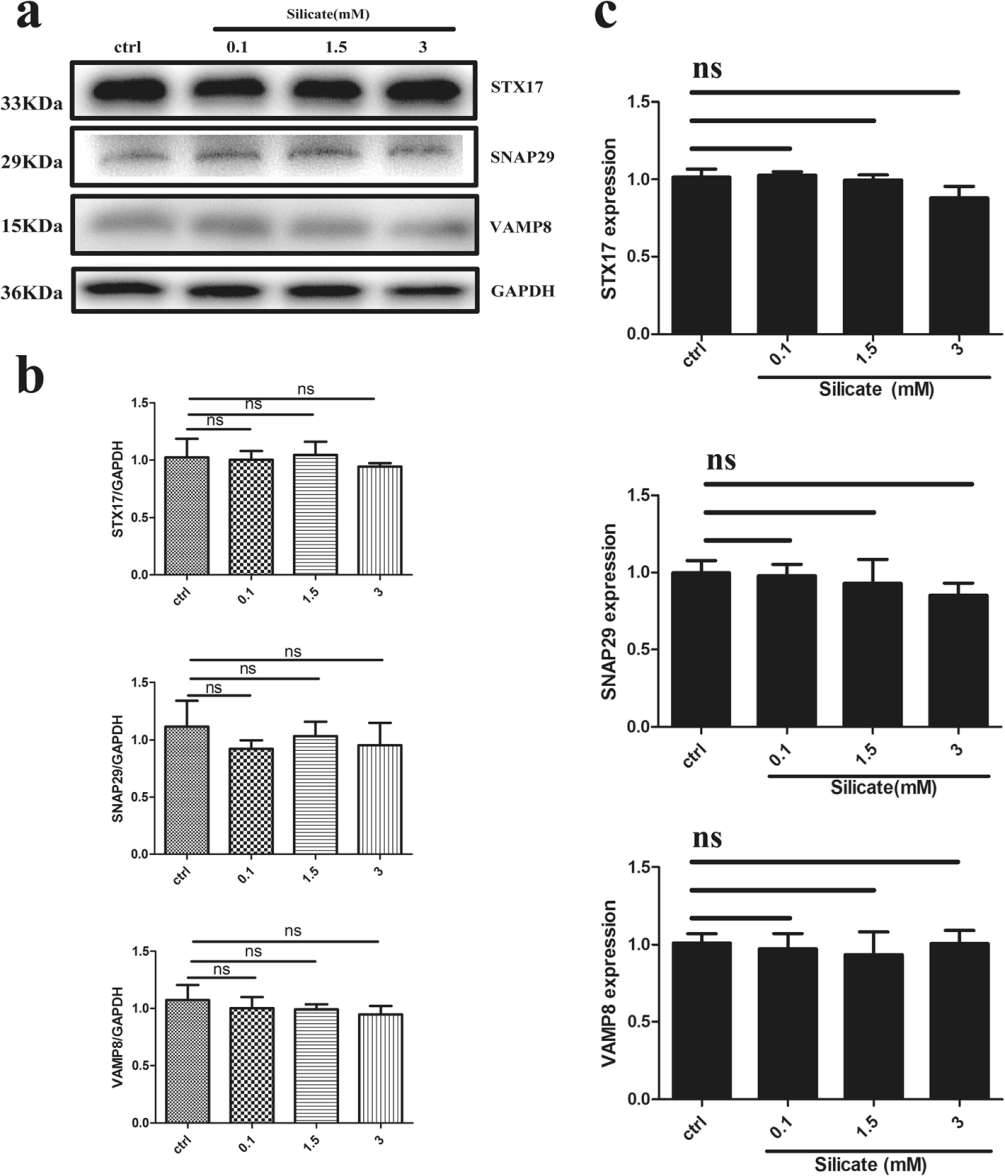
High concentration of silicate did not affect the expression of autophagosome-lysosome fusion related proteins of BMSCs. **a, b** Autophagosome-lysosome fusion-related proteins including SNAP29, STX17, and VAMP8 were determined by Western blot (*n* = 4). There was no significant difference after exposure to different doses of silicate for 24 h. **c** RT-qPCR further confirmed that there was no significant difference in the gene expression of SNAP29, STX17, and VAMP8 between groups (*n* = 4). (ns: no significance)

**Fig. 6:**
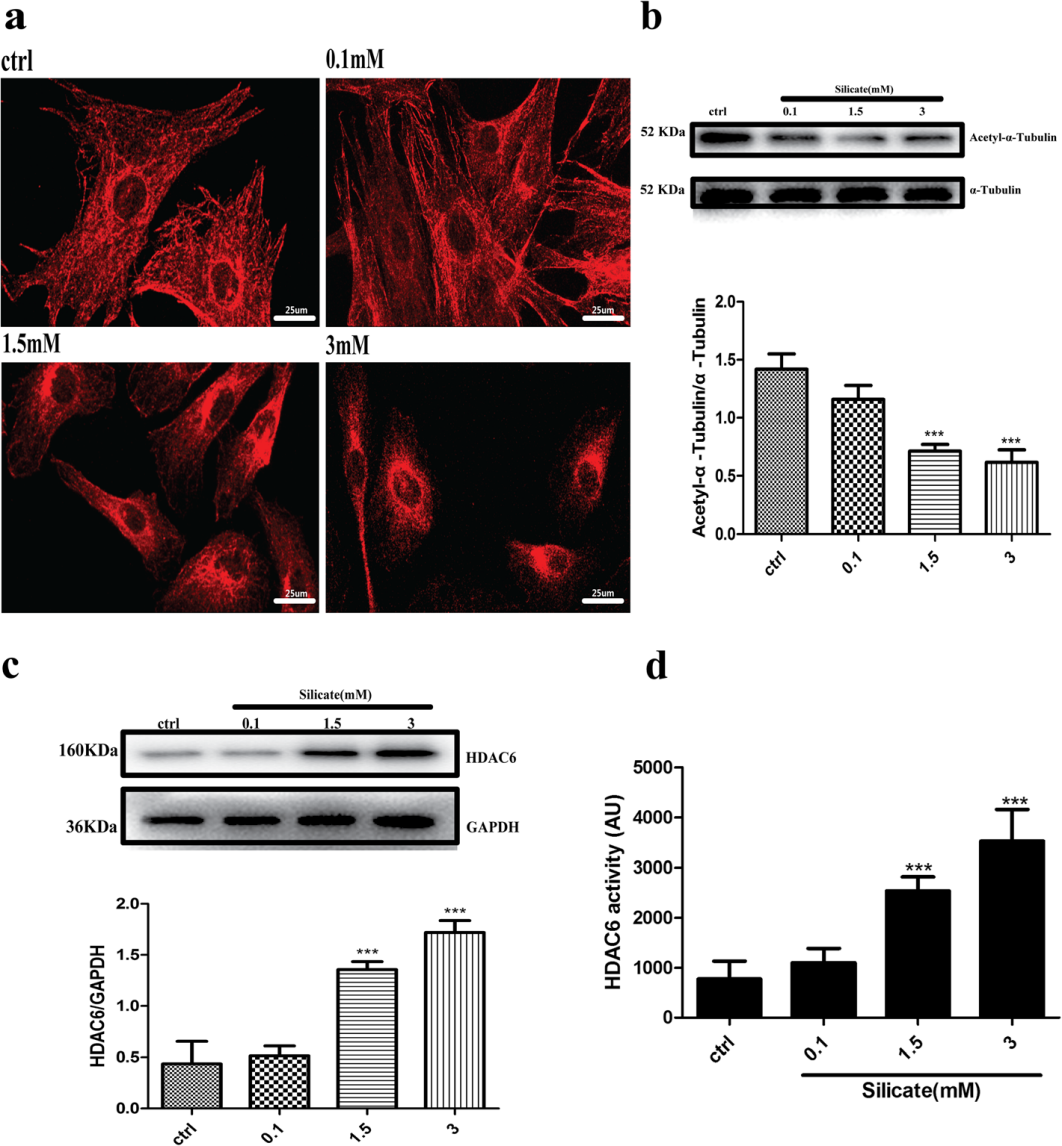
Silicate impaired microtubule stability via deacetylation of α-tubulin. **a** The structure of microtubules was evaluated with tubulin-tracker. As the concentration of silicate increased, the structure of the microtubules presented an obvious collapsed state with cell volume decrease and loss of bundle or reticular structure. **b** Western blot showed that acetylation of α-tubulin in BMSCs was decreased after exposure to silicate for 24 h (*n* = 4). **c, d** HDAC6, a specific enzyme responsible for regulating the acetylation process of α-tubulin, was evaluated via Western blot and HDAC6 activity assay (*n* = 4). The data indicated that the expression and activity of HDAC6 in the 1.5 mM and 3 mM silicate groups demonstrated a significant increase compared with that of the control group. (***p < 0.001)

To further confirm the reasons for the blockage of autophagic flux, the function of lysosomes, autophagosome-lysosome fusion-related proteins (SNAP29, STX17, VAMP8), and microtubule stability were detected. The lyso-tracker assay, which could lock acid lysosomes, was applied to evaluate the function of lysosomes, and the Baf A1 and CQ treatment groups were set as the positive control groups. The lysosome fluorescence intensity was distinctly suppressed in the Baf A1 group compared with that of the control group (*p* < 0.05), while in the CQ and silicate (3 mM) treatment groups, the lysosome fluorescence intensity was significantly increased compared with that of the control group; there was no statistical difference between the CQ group and silicate group (Fig. 4a, b). Moreover, the lysosome-related protein markers, including LAMP1 and LAMP2, were detected with Western blot after treatment with different concentrations of silicate (0, 0.1, 1.5, 3 mM). As shown in Fig. 4c, d, both LAMP1 and LAMP2 significantly increased in the groups treated with 1.5 mM and 3 mM silicate compared with that of the control group (*p* < 0.001 for LAMP1, *p* < 0.05 for LAMP2 in the 1.5 mM group, *p* < 0.01 for LAMP2 in the 3 mM group). Then, autophagosome-lysosome fusion-related proteins including SNAP29, STX17, and VAMP8 were determined by Western blot and RT-qPCR. There was no statistically significant difference between the silicate group and control group in the protein expression of SNAP29, STX17, and VAMP8 after 24 h of exposure to different concentrations of silicate (Fig. 5a, b). Gene detection via RT-qPCR further confirmed that high-dose silicate had no significant effect on the expression of SNAP29, STX17, and VAMP8 genes (Fig. 5c). Finally, the structure of the microtubules was evaluated with a tubulin-tracker (Fig. 6a). As the concentration of silicate increased, the structure of the microtubules presented an obvious collapsed state. On the one hand, the cell volume decreased significantly; on the other hand, the microtubule lost its bundle or reticular structure, especially in the 3 mM silicate group. Acetylated tubulin is essential for the stabilization of microtubules and facilitation of intracellular trafficking [26, 27]; therefore, the rate of acetyl-α-tubulin/α-tubulin was further detected via Western blot. As shown in Fig. 6b, the acetyl-α-tubulin protein was significantly downregulated after treatment with a high dose of silicate (1.5 mM and 3 mM groups) compared with that of the control group (*p* < 0.001). Furthermore, HDAC6, a specific enzyme responsible for regulating the acetylation process of α-tubulin [28, 29], was also evaluated via Western blot and HDAC6 activity assay. As shown in Fig. 6c, d, the expression and activity of HDAC6 in the 1.5 mM and 3 mM silicate groups demonstrated a significant increase compared with that of the control group (*p* < 0.001).

### Inhibition of HDAC6 activity restored microtubule stability and autophagic activity

**Fig. 7:**
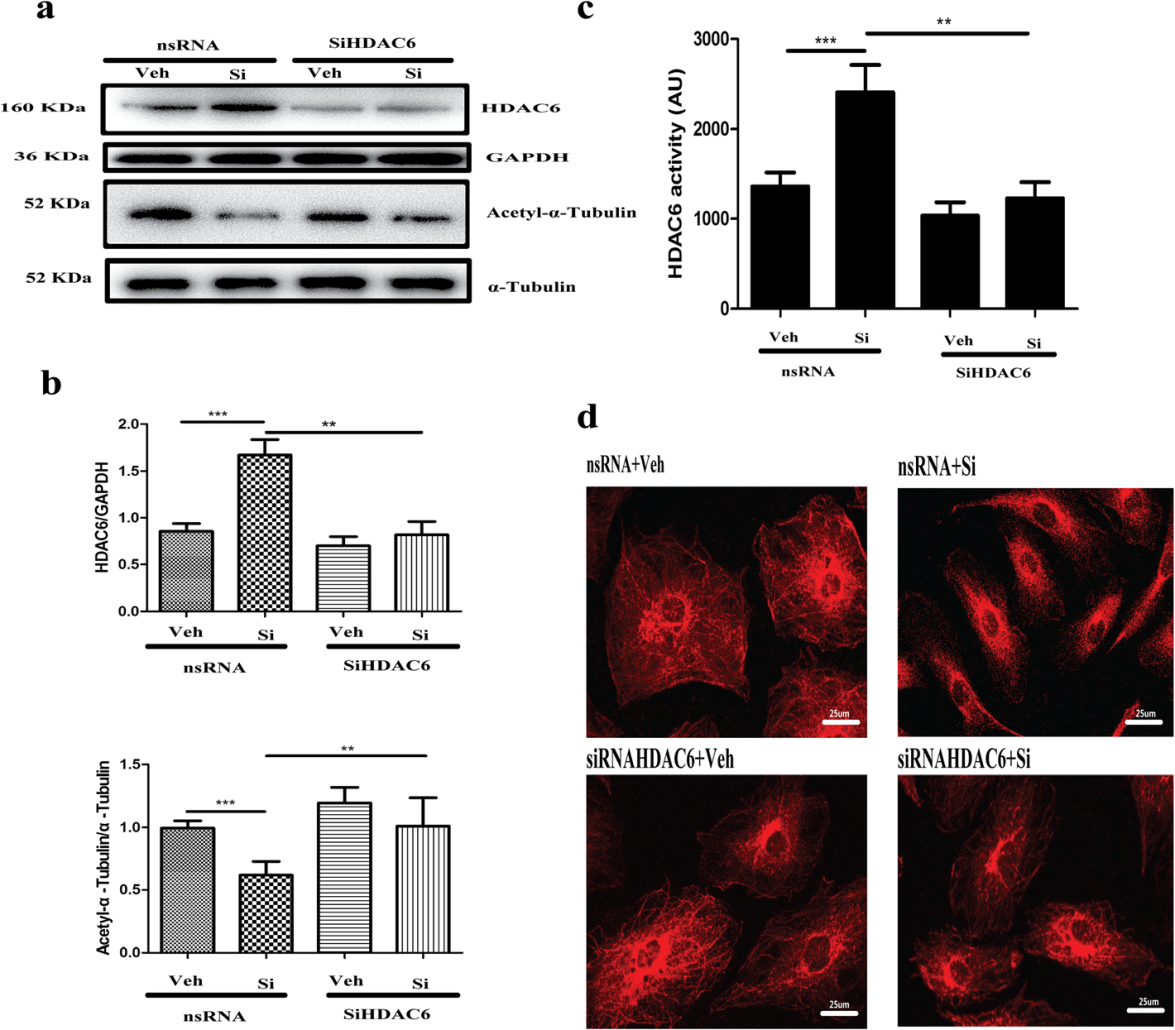
Inhibition of HDAC6 activity restored microtubule stability after silicate exposure. HDAC6 expression was silenced via siHDAC6 transfection (50 nM) and subsequently exposed to a high dose of silicate (3 mM). **a, b, c** HDAC6 expression and activity were significantly downregulated compared with that of the control group after transfection with siHDAC6 from Western blot detection and HDAC6 activity assay, and the suppression of HDAC6 significantly increased the expression of acetyl-α-tubulin with Western blot detection (*n* = 4). **d** Collapsed state and bundle or reticular structure of microtubules, caused by high-dose silicate, was partially restored via the suppression of HDAC6. (**p < 0.01, ***p < 0.001)

**Fig. 8:**
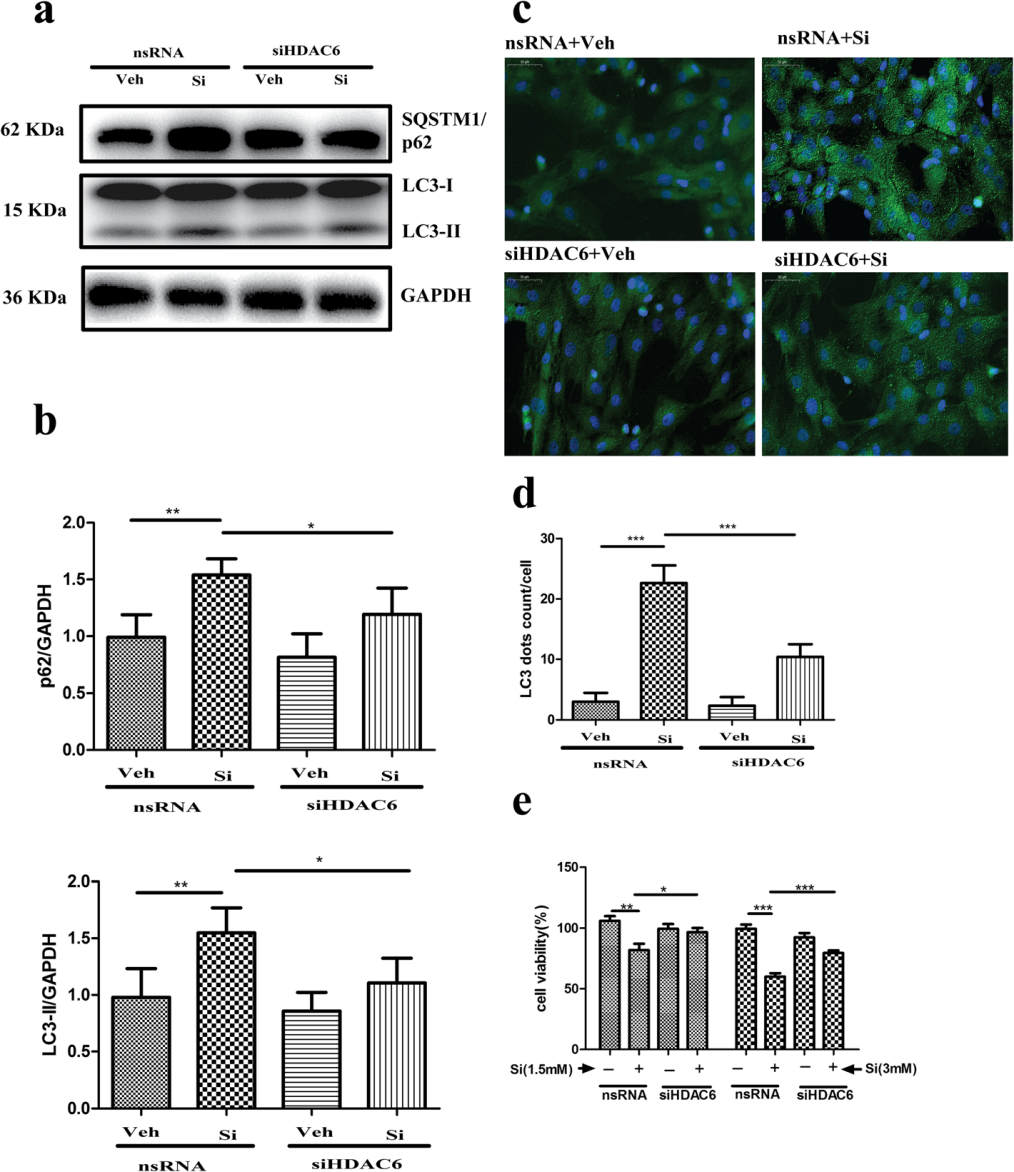
Inhibition of HDAC6 activity restored autophagic activity and improved cell viability after silicate exposure. **a, b** Autophagic flux-related proteins including p62 and LC3-II were identified by Western blot after transfection with siHDAC6 (*n* = 4). Data analysis showed that the expression of p62 and LC3-II after high-dose silicate treatment was partially downregulated via HDAC6 interference compared with that of the control group. **c, d** Autophagosome (LC3 dots) accumulation caused by high-dose silicate was partially improved with siHDAC6 treatment (*n* = 3, 10 random fields per sample). **e** CCK-8 assay was applied to evaluate cell viability after siHADC6 treatment. As the data show, the downregulation of cell viability caused by 1.5 mM and 3 mM silicate was significantly improved after siHDAC6 treatment (*n* = 4). (*p < 0.05, **p < 0.01, ***p < 0.001)

To confirm the relationship of HDAC6 activity with microtubule stability and autophagic flux, HDAC6 expression was silenced via siHDAC6 transfection. The expression of HDAC6 mRNA declined 85.8% (nsRNA = 50.49 ± 13.55 vs SiHDAC6 = 7.19 ± 3.20) when the cells were treated with 50 nM siRNA. HDAC6 activity was significantly downregulated compared with that of the control group after transfection with siHDAC6 from Western blot detection and HDAC6 activity assay (*p* < 0.01; Fig. 7a–c). Simultaneously, the expression of acetyl-α-tubulin protein was detected via Western blot. As shown in Fig. 7a, b, the suppression of HDAC6 significantly increased the expression of acetyl-α-tubulin (*p* < 0.01); moreover, the structure of the microtubules was also evaluated with a tubulin-tracker. As shown in Fig. 7d, the collapsed state of BMSCs, which was caused by high-dose silicate, was partially restored via the suppression of HDAC6. The autophagic flux-related proteins including p62 and LC3-II were identified after transfection with siHDAC6. As shown in Fig. 8a, b, the expression of p62 and LC3-II after high-dose silicate treatment was partially downregulated via HDAC6 interference compared with that of the control group (*p* < 0.05). The immunofluorescence detection of LC3 further confirmed that autophagosome (LC3 dots) accumulation caused by high-dose silicate was partially improved with siHDAC6 treatment (*p* < 0.001; Fig. 8c, d). Furthermore, CCK-8 assay was applied to evaluate cell viability after siHADC6 treatment. As shown in Fig. 8e, the downregulation of cell viability caused by 1.5 mM and 3 mM silicate was significantly improved after siHDAC6 treatment (*p* < 0.05 in the 1.5 mM group and *p* < 0.001 in the 3 mM group).

## Discussion

Bone formation mainly involves two major processes: osteogenesis and angiogenesis. BMSCs are indispensable in the process of bone formation and play an accelerative role in promoting angiogenesis [30]. Biocompatibility of biomaterials with the host microenvironment plays a crucial role in maintaining the adhesion, proliferation, and differentiation of BMSCs. Silicon is a kind of trace element in the human body and is especially important for bone growth and development [31]. Extensive studies have confirmed that silicon-based biomaterials can not only promote osteogenesis formation but also stimulate angiogenesis via BMSC interaction [4, 32]. In recent years, an increasing number of researchers have paid attention to the cytotoxic mechanisms of silicon-doped materials; whether silicon nanomaterials or high concentration of free silicate, both can affect the cell viability to some extent [33]. In this study, it was found that treating BMSCs with a high concentration of soluble silicate could affect the viability of BMSCs and cause the interruption of autophagic flux of BMSCs, and the interruption of autophagic flux was related to the destabilization of microtubule structure. Furthermore, microtubule destabilization caused by silicate was induced by HDAC6 activation via α-tubulin histone deacetylation.

Autophagy is a self-protective mechanism of cells under stress. The interruption of autophagic flux can further cause a large accumulation of autophagosomes, leading to an imbalance of cell homeostasis. On the one hand, damaged organelles inside cells cannot be removed, especially mitochondria and endoplasmic reticulum, which can directly trigger apoptosis; on the other hand, the interruption of the cell energy cycle leads to an energy crisis [34]. In this study, it was found that a high concentration of silicate can lead to a simultaneous increase of LC3-II and p62; LC3-II is the marker protein of autophagosomes, while p62 protein can bridge the binding of ubiquitination protein and LC3 to promote clearance of the targeted ubiquitination protein through autophagic flux; the simultaneous increase of both often indicates the interruption of autophagy flux [35]. Furthermore, we detected the LC3-II and p62 expression with LC3 turnover test via CQ and Baf A1 application. The data confirmed that a high concentration of silicate could lead to disruption of the autophagic flux (>1.5 mM), causing a large accumulation of autophagosomes via LC3 fluorescence detection. Accordingly, the cell viability test found that with a further increase in the high concentration of silicate, the intracellular silicon increased, and cell viability gradually decreased, suggesting that the interruption of autophagic flux may affect cell viability [36].

Autophagic flux is the process of the formation of autophagosomes within cells and eventual fusion with lysosomes. The regulation of autophagic flux involves various molecules and microstructures, among which the function of the lysosome is of vital importance and is the key point for the smooth flux of autophagy. Lyso-tracker is a lysosomal fluorescent probe that can selectively remain in acidic lysosomes, thus achieving specific fluorescent labeling of living cell lysosomes [37]. Our study indicated that a high concentration of silicate did not affect the acidic environment in lysosomes; moreover, CQ and Baf A1 were set as the positive control groups, and the study further confirmed that high-concentration silicate may interfere with autophagy flux in a way similar to CQ. Mauthe et al. found that the autophagic flux blocking mechanism of CQ and Baf A1 may not be the same: Baf A1 blocks autophagic flux mainly by altering lysosomal function, whereas CQ blocks the activity of autophagic flux by affecting membrane fusion between autophagosomes and lysosomes [38], which further suggests that a high concentration of soluble silicate may disrupt autophagic flux by affecting membrane fusion. In addition, we found that the expression of LAMP1 and LAMP2, two lysosomal membrane proteins [39], was increased after exposure to silicate. The inhibition of autophagy may promote lysosomal synthesis through a feedback mechanism that needs further research. We further investigated the protein and gene expression of membrane fusion assistance proteins including SNAP29, VAMP8, and STX17 [40]. The results showed that the silicate did not affect the expression of these proteins. However, it is still uncertain whether silicate can interfere with the protein interaction of different membrane fusion assistance proteins; this topic needs further study.

Microtubule structure plays an important role as a bridge in the combination of autophagosomes and lysosomes. In recent years, numerous studies have identified that hyperacetylation of α-tubulin is necessary for the stimulation of autophagy. It was found that a decrease in α-tubulin acetylation induced by an acidic environment inhibited autophagic activity and induced rat cardiomyocyte injury [41–43]. Wang et al. reported that rats with silicosis caused by silica solution (50 mg/rat, 1 ml) via trachea instillation displayed a significant decrease in the expression of acetyl-α-tubulin. This loss of deacetylation was associated with activation of HDAC6 [44]; thus, we further detected changes in the microtubule structure as well as hyperacetylation of α-tubulin. We demonstrated that the microtubule structure was obviously disordered after the intervention of high-concentration silicate in BMSCs. It was found that acetyl-α-tubulin, which maintained microtubule homeostasis, was significantly reduced, which also preliminarily suggested that a high concentration of silicate might affect the acetylation of microtubule proteins, further leading to the disintegration of microtubules and thereby blocking the trafficking of autophagosomes to lysosomes. α-Tubulin acetylated modification was regulated by HDAC6 [45, 46], and our further research also confirmed that a high concentration of silicate can promote the expression of HDAC6. After using siRNA to interfere with HDAC6 expression, α-tubulin acetylation was enhanced significantly compared with silicate treatment alone, and the microtubule structure was also partially recovered with more beam-like divergent structures. We then verified that autophagic flux was partially restored with reduced autophagosome accumulation via inhibiting HDAC6 expression. Simultaneously, cell viability increased significantly via HDAC6 suppression after high-dose silicate exposure, which indicated that the combination with siHDAC6 may provide a feasible way to protect BMSCs from the toxicity of high silicate exposure.

**Fig. 9:**
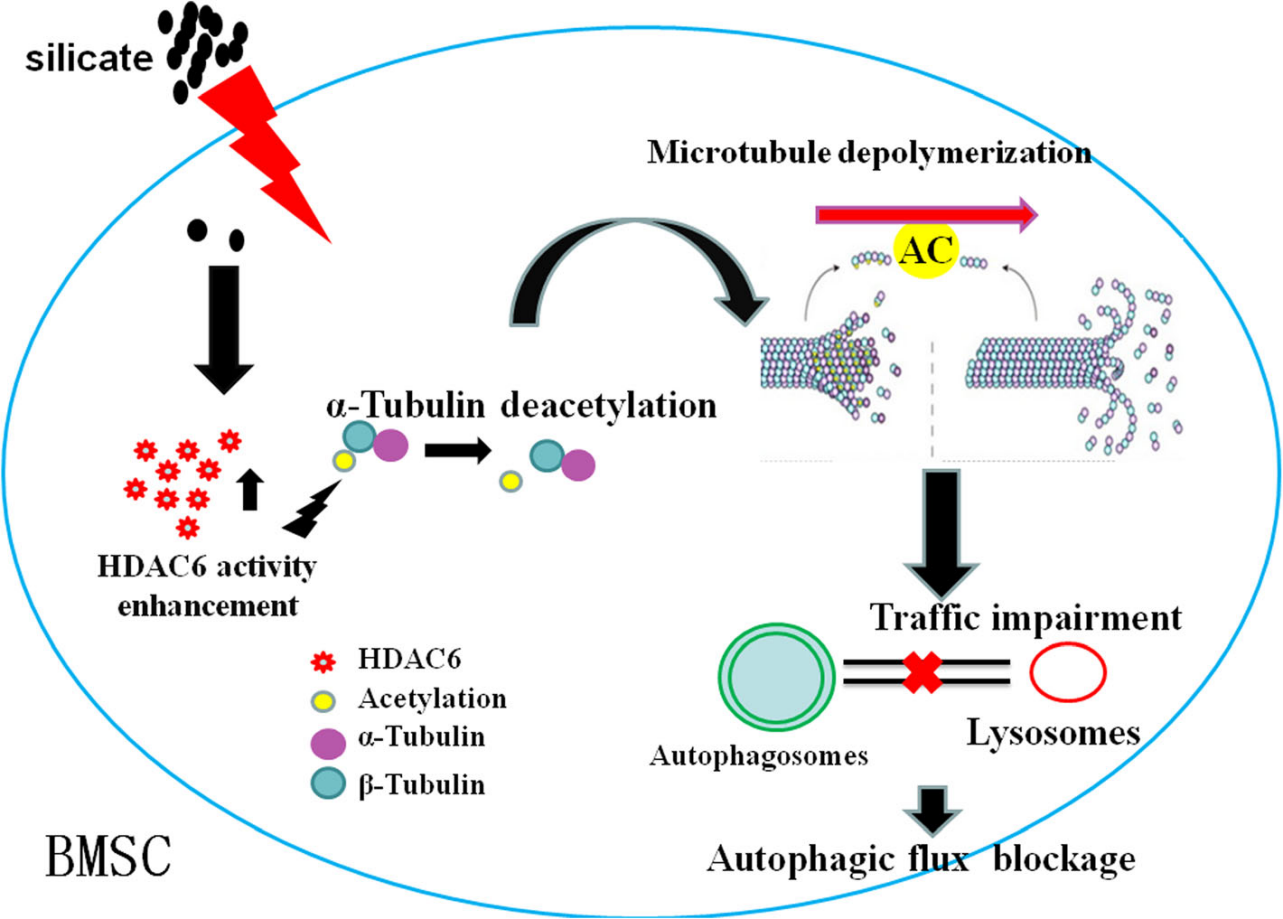
Schematic diagram of silicate-induced blockage of autophagic flux. The silicate enters the BMSCs and induces the enhancement of HDAC6 activity and expression. HDAC6 further leads to the deacetylation of α-tubulin, resulting in the depolymerization of the microtubule. The autophagosomes cannot be transported to fuse with lysosomes as the microtubule disruption, which eventually leads to the blockage of autophagic flux

Silicate, as a representative of silicon ions, has been widely used in bone regeneration engineering. However, the concentration of silicate used in biomaterials is often several times or even hundreds of times higher than the normal range in human serum (85–130 μg/l) [13, 14]. This study, from the perspective of autophagy, redefines the safety range of silicate in biomaterials research for BMSCs. In brief, a high concentration of silicate intra-cell can enhance the activity of HDAC6; subsequently, microtubule depolymerized due to the reduction of acetyl-α-tubulin, and silicate may interfere with autophagic flux activity via disturbing the stability of microtubule when the concentration exceeds 1.5 mM (Fig. 9). Autophagy generally occurs earlier than cell apoptosis, which preliminarily suggests that the reasonable application of the autophagic flux test for biomaterials is of great importance. For BMSC-related studies, biomaterials containing silicon shall not exceed a concentration of 1.5 mM, and the autophagic flux test should be set as a reference indicator for biocompatibility and safety detection. Of course, the reasonable application range of different biomaterials or different organisms should be treated separately in vivo and in vitro, and this requires further exploration and discussion.

## Conclusions

Our study proves that a high concentration of silicate can affect the viability and cause the blockage of autophagic flux in BMSCs, and the blockage of autophagic flux was related to the destabilization of the microtubule structure. Furthermore, microtubule destabilization caused by silicate was induced by HDAC6 activation via α-tubulin histone deacetylation. This study from the perspective of autophagy redefines the safety range of silicate in biomaterials research for BMSCs; silicate may interfere with autophagic flux activity when the concentration exceeds 1.5 mM.

## Supplementary information

**Additional file 1. **Cell viability changes after treatment with different concentrations of silicate at 6 h and 72 h. **(a)** Cell viability decreased significantly in silicate concentration higher than 3 mM compared with that of control group after 6 h exposure to silicate (*n* = 6). **(b)** Cell viability decreased significantly in silicate concentration higher than 1.5 mM compared with that of control group after 72 h exposure to silicate (**n** = 6).

**Additional file 2. **Cell morphologic changes and autophagic proteins expression after 3 weeks exposure to silicate. **(a-c)** BMSCs were treated with long-term (3 weeks) of 0.1 mM and 0.5 mM silicate. Compared with the control group, the silicate did not show obvious cytotoxicity, the cells did not float or break as they did after treatment with high concentration of silicate, and some nodular changes can be observed during the cells proliferation (Arrow). **(d-e)** we analyzed the expression of LC3-II and p62 proteins via western-blot after a long-term culture of BMSCs, and it was also found that there was no statistical difference in protein changes (*n* = 4).

## Data Availability

Original data that support the findings of this study are available from the corresponding author upon reasonable request.

## Abbreviations

Baf A1: Bafilomycin A1
BMSCs: Bone mesenchymal stem cells
CQ: Chloroquine
HDAC6: Histone deacetylase 6
Snap29: Synaptosome associated protein 29
STX17: Syntaxin 17
Vamp8: Vesicle-associated membrane protein 8
