# Supplementary figures and images for: Microtubule destabilization caused by silicate via HDAC6 activation contributes to autophagic dysfunction in bone mesenchymal stem cells
Source: Stem Cell Res Ther. 2019 Nov 27;10:351. doi: 10.1186/s13287-019-1441-4 (PMC6880487; doi:10.1186/s13287-019-1441-4)

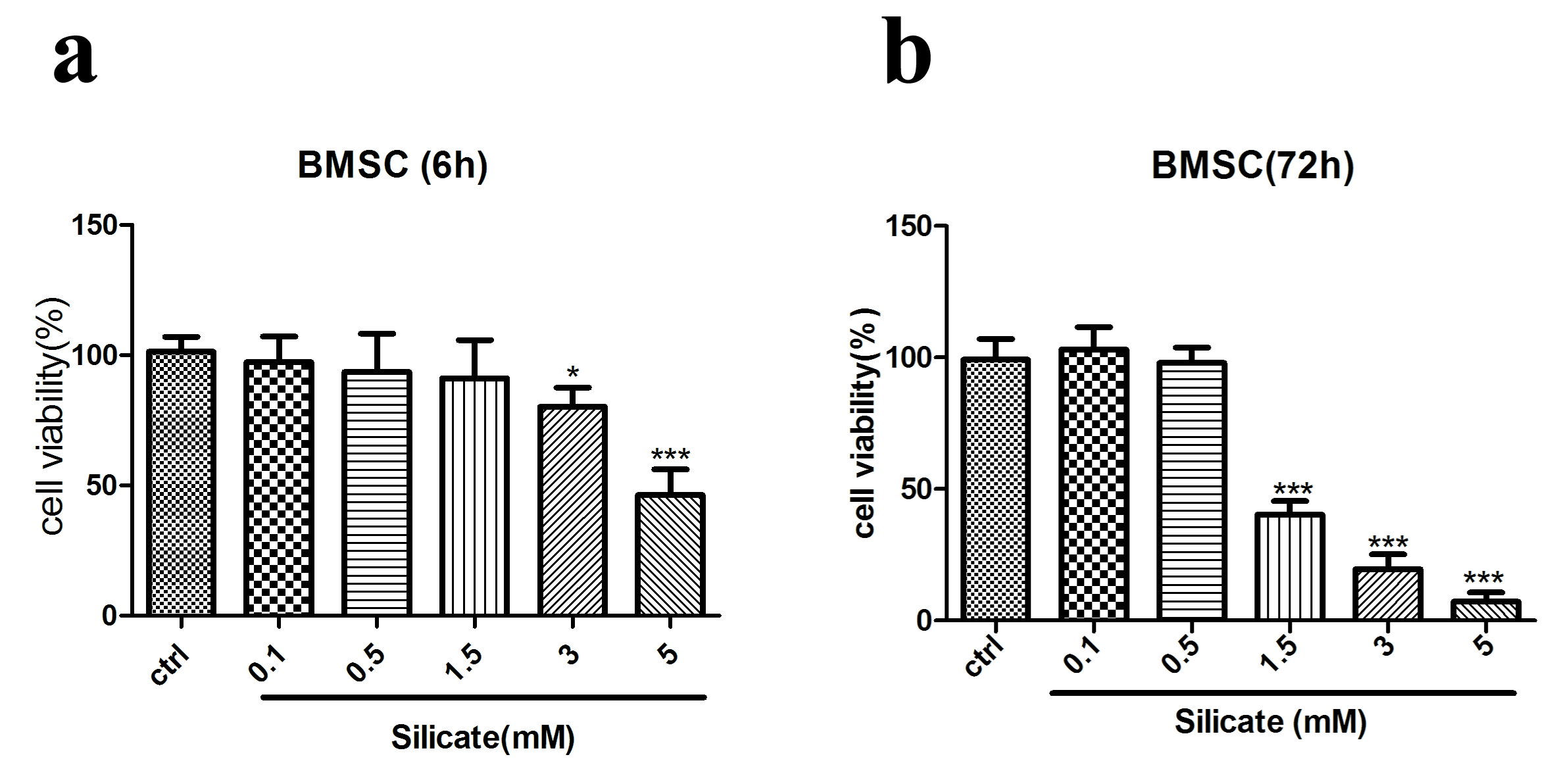

Supplement: Supplementary file 1 — Additional file 1. Cell viability changes after treatment with different concentrations of silicate at 6 h and 72 h. (a) Cell viability decreased significantly in silicate concentration higher than 3 mM compared with that of control group after 6 h exposure to silicate (n = 6). (b) Cell viability decreased significantly in silicate concentration higher than 1.5 mM compared with that of control group after 72 h exposure to silicate (n = 6). [file 13287_2019_1441_MOESM1_ESM.tif]

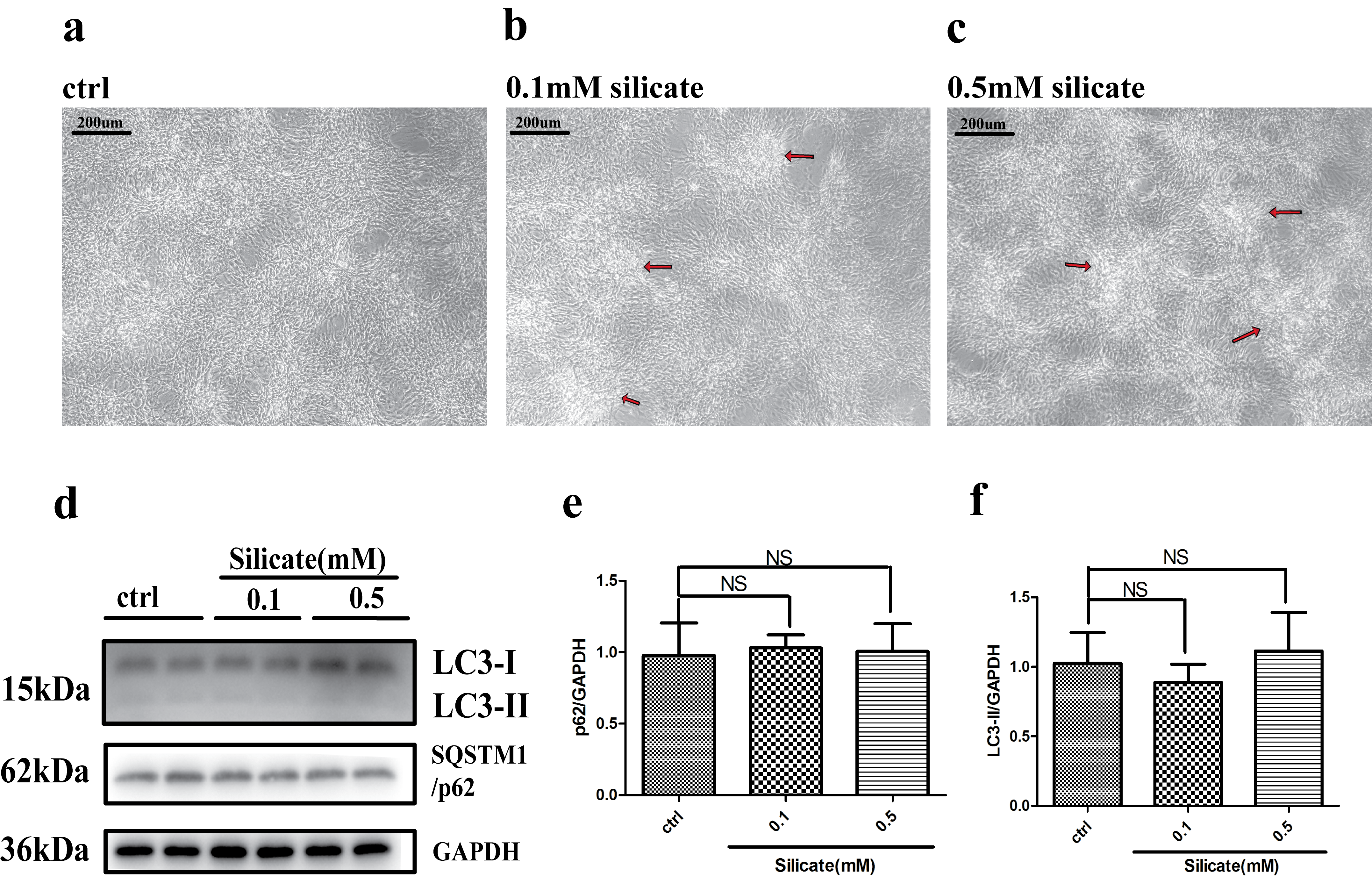

Supplement: Supplementary file 2 — Additional file 2. Cell morphologic changes and autophagic proteins expression after 3 weeks exposure to silicate. (a-c) BMSCs were treated with long-term (3 weeks) of 0.1 mM and 0.5 mM silicate. Compared with the control group, the silicate did not show obvious cytotoxicity, the cells did not float or break as they did after treatment with high concentration of silicate, and some nodular changes can be observed during the cells proliferation (Arrow). (d-e) we analyzed the expression of LC3-II and p62 proteins via western-blot after a long-term culture of BMSCs, and it was also found that there was no statistical difference in protein changes (n = 4). [file 13287_2019_1441_MOESM2_ESM.tif]
